# Proteomic and metabolomic analysis of *Nicotiana benthamiana* under dark stress

**DOI:** 10.1002/2211-5463.13331

**Published:** 2021-12-16

**Authors:** Juan‐Juan Shen, Qian‐Si Chen, Ze‐Feng Li, Qing‐Xia Zheng, Ya‐Long Xu, Hui‐Na Zhou, Hong‐Yan Mao, Qi Shen, Ping‐Ping Liu

**Affiliations:** ^1^ College of Chemistry Zhengzhou University Zhengzhou China; ^2^ Chemistry Research Institution of Henan Academy of Sciences Zhengzhou China; ^3^ Zhengzhou Tobacco Research Institute of CNTC Zhengzhou China

**Keywords:** autophagy, dark stress, metabolism, *Nicotiana benthamiana*, proteomic, weighted gene coexpression network analysis

## Abstract

Exposure to extended periods of darkness is a common source of abiotic stress that significantly affects plant growth and development. To understand how *Nicotiana benthamiana* responds to dark stress, the proteomes and metabolomes of leaves treated with darkness were studied. In total, 5763 proteins and 165 primary metabolites were identified following dark treatment. Additionally, the expression of autophagy‐related gene (ATG) proteins was transiently upregulated. Weighted gene coexpression network analysis (WGCNA) was utilized to find the protein modules associated with the response to dark stress. A total of four coexpression modules were obtained. The results indicated that heat‐shock protein (HSP70), SnRK1‐interacting protein 1, 2A phosphatase‐associated protein of 46 kDa (Tap46), and glutamate dehydrogenase (GDH) might play crucial roles in *N. benthamiana*’s response to dark stress. Furthermore, a protein–protein interaction (PPI) network was constructed and top‐degreed proteins were predicted to identify potential key factors in the response to dark stress. These proteins include isopropylmalate isomerase (IPMI), eukaryotic elongation factor 5A (ELF5A), and ribosomal protein 5A (RPS5A). Finally, metabolic analysis suggested that some amino acids and sugars were involved in the dark‐responsive pathways. Thus, these results provide a new avenue for understanding the defensive mechanism against dark stress at the protein and metabolic levels in *N*. *benthamiana*.

## Graphical abstract

Exposure to prolonged periods of darkness can affect plant growth and development. In this study, the response of *Nicotiana benthamiana* to dark stress was investigated by analyzing changes to the proteome and metabolome across 13 time points. A number of hub proteins were filtered through weighted gene coexpression network analysis to identify potential key proteins involved in the dark stress response.

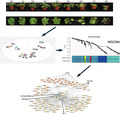

**Abbreviations**
ABA: abscisic acid
ACN: acetonitrile
Akt: AKT serine/threonine kinase 1
ANOVA: analysis of variance
AT2G43090: isopropylmalate isomerase small subunit 1
ATGs: autophagy‐related genes
ATPA: ATP synthase CF1 alpha subunit
ELF5A: eukaryotic elongation factor 5A
FDR: false discovery rate
GC: gas chromatography
GDH: glutamate dehydrogenase
HCA: hierarchical clustering alg
HPLC: high‐pressure liquid chromatography
HSP: heat‐shock protein
IDA: information‐dependent acquisition
IIL1: isopropylmalate isomerase large subunit 1
IPMI: isopropylmalate isomerase
LC: liquid chromatography
LC3II: mictotubule‐associated protein light chain 3II
MS: mass spectrometry
mTOR: mammalian target of rapamycin
PCA: principal component analysis
PP2A: protein phosphatase 2A
PPI: protein–protein interaction
PSAF: photosystem I subunit F
RNA: ribonucleic acid
ROS: reactive oxygen species
RPL24: ribosomal protein L24
RPL5A: ribosomal protein L5A
RPS5A: ribosomal protein 5A
RT‐PCR: reverse‐transcription polymerase chain reaction
Rubisco: ribulose bisphosphate carboxylase oxygenase
SA: salicylic acid
SD: standard deviation
SnRK1: SNF1‐related kinase 1
SWATH‐MS: sequential windowed data‐independent acquisition of the total high‐resolution mass spectra
Tap46: 2A phosphatase associated protein of 46 kDa
TEM: transmission electron microscope
TLP18.3: thylakoid lumen 18.3 kDa protein.
TOR: target of rapamycin
TORC1: target of rapamycin complex 1
WGCNA: weighted gene coexpression network analysis

The direct effect of illumination on plants is to carry out photosynthesis, so that organic material could be produced for plants to store energy. Illumination can also be used as a signal factor to regulate plant growth and development, including all stages of plant growth [1]. Lack of illumination is likely to lead to carbohydrate deficiency in plant cells [2]. The synthesis of chlorophyll is dependent on illumination, and the content and activity of pigment complex proteins are affected by illumination intensity. Chlorophyll synthesis is inhibited, and the expression of related pigment complex proteins is downregulated under dark conditions [3]. Duba and Carpenter [4] found that the leaves became larger and thinner; the net photosynthetic rate decreased of American sycamore clone grown in shade. Wada [5] identified the number and size of chloroplasts significantly reduced in darkened leaves of wild‐type *Arabidopsis*, and the protein content in chloroplasts decreased. Dark stress also induces autophagy to recycle cytoplasmic contents in order to provide energy requirements [3, 6, 7, 8]. In order to maintain respiration in darkness, protein and lipid catabolism can replace carbohydrate metabolism. Damaged proteins, organelles, and other degraded materials are enveloped by double‐membrane autophagosomes and transferred to vacuole [9, 10, 11, 12]. The outer membrane of the autophagosome fuses with the vacuole membrane, and the autophagosome is degraded in the vacuole to release amino acids and carbohydrates for recycling [13, 14]. This process requires not only ATG proteins but also proteins that can mediate and regulate the process of autophagy [15].

There are several ways to achieve the dark treatment for plants, such as the entire plants are put in dark condition for cultivation, the mature leaves are cut off from plants and incubated in a medium placed in dark environment, and the plant leaves wrapped in tin foil are individually shaded [5, 16, 17]. *N. benthamiana* is an important model system which is usually used to study gene regulation in response to stress. Therefore, it is of great significance to study the performances of *N. benthamiana* under dark stress.

Following the rapid progress in liquid chromatography/mass spectrometry (LC/MS) technology, sequential windowed data‐independent acquisition of the total high‐resolution mass spectra (SWATH‐MS) is increasingly used for proteome analysis to identify proteins and pathways that are vital to stress response [18, 19]. At present, studies on dark stress are almost based on gene level [3, 20]. We aimed to reveal the changes that take place in the proteome of *N. benthamiana* in response to dark stress by SWATH‐MS method coupled with bioinformatic analyses. In addition to protein regulatory networks, plants also have a variety of highly regulated metabolic networks which play crucial roles in the growth process under dark treatment. For example, amino acids and sugars are not only energy substances but also signal molecules to participate in the intracellular signal transduction process and responding to stress [21, 22]. Therefore, multiomics analysis could present more comprehensive information. McLoughlin et al. [23] integrated proteomic, transcriptomic, and metabolomics analyses to research maize autophagy mutants subjected to carbon starvation induced by darkness, identified numerous autophagy‐responsive proteins, and revealed metabolic alterations during dark stress. Havé et al. [24] applied a multiomics approach to uncover how low‐nutrient conditions affect the changes in endoplasmic reticulum and lipid composition of *Arabidopsis atg5* mutants.

Herein, our goals were to explore the changes of proteome and metabolome of *N. benthamiana* in response to dark stress. WGCNA was used to establish modules in order to find proteins which respond to dark stress. Through the predicted‐PPI network, it was speculated that proteins IPMI, ELF5A, and RPS5A may play important roles in dark treatment by participating in the autophagy regulatory process. In addition, energy metabolism and hormones appear to play a significant role in the mechanism of *N. benthamiana* responding to dark stress.

## Methods

### Plant growth and dark treatment

Fifty‐day‐old *N. benthamiana* plants grown in soil were divided into the control group and the dark treatment group. Control group plants at time 0 h were placed in a normal environment with a circadian rhythm of 16 h of light (24 **°**C) and 8 h of darkness (20 **°**C), and plants were watered every other day. The dark‐treated plants had the same conditions as those of the control group except light. They were kept in a dark room up to specific time points along a time gradient (0 h (control), 8 h, 16 h, 24 h, 32 h, 40 h, 48 h, 3 days, 4 days, 5 days, 6 days, 7 days, and 8 days) [3]. The entire leaves of four plants were harvested at each time point during the dark treatment. After harvesting, the leaves were immediately frozen in liquid nitrogen and stored at −80 °C. All experiments were repeated three times.

### Quantitative RT‐PCR analysis

The EASYspin Plus Plant RNA Kit (AidLab, Beijing, China) was used to purify the total ribonucleic acid (RNA) of samples from each time point according to the instructions. The RNA from each sample was used in reverse transcription with the primescript RT reagent Kit with gDNA Eraser (Takara, Kyoto, Japan). Specific primers designed for the real‐time quantitative RT‐PCR of ATG8i and TOR were as follows: 5' ATATAATCGCCAAATATCCTGATC 3' (upstream primer) and 5' TGTTGTTTGAGGCAAGGTGTTA 3' (downstream primer), 5' AAGCCCACGCTTTATTTGCG 3' (upstream primer) and5' TTCAACTTGCGCCTACCCTT 3' (downstream primer), respectively (Appendix S1). PCRs were performed using the LightCycler^®^ 96 real‐time PCR detection system (Roche, Basel, Switzerland).

### HPLC analysis of pigment content

20 mg dried sample powder was extracted with 1.5 mL extract solution (90% acetone), treated with ultrasonic for 1 h, and centrifuged for 10 min. The supernatant was collected and analyzed by high‐pressure liquid chromatography (HPLC).

Agilent 1260 HPLC (Agilent, Santa Clara, CA, USA) connected to a C18 column (3.9 mm × 150 mm, Waters, Milford, MA, USA) were used. The flow gradient was as follows (buffer A: isopropanol; buffer B: 80% acetonitrile (ACN)): buffer B from 100% to 0% in 0–39 min. The flow rate was 0.5 mL·min^−1^. The ultraviolet wavelengths of detection were at 428 and 448 nm.

### TEM analysis

Plant leaves were cut into thin slices, fixed with 2.5% glutaraldehyde for 2 h and 1% osmic acid for 4 h, respectively. The slices were dehydrated with ethanol and embedded in the resin at 37 °C for 12 h after washing with phosphate buffer. Then, the resin was sliced and dyed with 2% (w/v) uranium dioxide acetate for 20 min followed by lead citrate for 10 min. TEM HT 7700 (HITACHI, Tokyo, Japan) was used to observe and photograph the cells.

### GC/MS sample preparation and analysis

The method of Lu [25] was used for gas chromatography/mass spectrometry (GC–MS) analysis with some modification. 20 mg of dry leaves and 1.5 mL extraction solvent (isopropyl alcohol/ACN/water:3/3/2 (v/v/v)) with tridecanoic acid as internal standard were mixed by ultrasound for 1 h. After centrifugation (21 130 *g*, 10 min), 500 μL of the supernatant was collected and dried, then derivatized at 37 °C for 90 min in 100 μL of 20 mg·mL^−1^ methoxyamine pyridine (Merck, Darmstadt, Germany) solution and 100 μL of *N*‐methyl‐*N*‐(trimethysilyl) trifluoroacetamide (Merck) for 60 min at 60 °C, respectively.

An Agilent 6890 Series GC system combined with an Agilent 5975 series mass selective detector were used. An Agilent DB‐5MS column (0.25 μm, 0.25 mm × 30 m) was chosen. Flow rate was set at 1.2 mL·min^−1^, 300 °C as the injection temperature, 230 °C as source temperature, and oven temperature programmed at 70 °C for the first 4 min and then increased at 5 °C·min^−1^ to 310 °C for 15 min. The scan range was set to 33–500 *m/z* in full scan mode. The data were quantitatively processed with autogcmsdataanal software [26]; the NIST library (version 2.3) was used for qualitative analysis of metabolites.

### LC/MS analysis of plant hormones and flavonoids

25 mg of dry *N. benthamiana* leaves power were dissolved in 0.75 mL of cooled methanol water extract solution (4 : 1, v/v). Samples were ultrasonic for 30 min at room temperature and kept at 4 °C overnight. The supernatant was collected, and ten times concentrated. Hormones and flavonoids analyses were performed using an LC/MS system (LC, Agilent 1260 HPLC system; MS, Agilent6490 triple quadrupole).

SB C18 column (2.1 mm × 100 mm, 1.8 μm, Agilent) was chosen for the detection of hormones. The flow rate was 0.3 mL·min^−1^. Multireaction monitoring (MRM) mode was adopted. Buffer A (0.1% formic acid in water) and buffer B (0.1% formic acid methanol solution) were as the mobile phases, and the gradient was set as: buffer B increased from 5% to 48% in 0–0.2 min; 57% in 7.0 min; 96% in 7.5 min; 100% in 10.0 min.

BEH Phenyl (2.1 mm × 150 mm, 1.7 μm, Waters) was chosen for the detection of flavonoids. The flow rate was 0.3 mL·min^−1^. There were 10 compounds monitored by MRM mode. The flow gradient was as follows (buffer A: 0.1% formic acid in water; buffer B: 0.1% formic acid ACN): buffer B from 5% to 15% in 0–2 min; 15% in 10 min; 100% in 15 min.

### Determination of starch by continuous flow method

The starch content was determined according to the tobacco industry standards of the People's Republic of China (Standard no: YC/T 216‐2013). 25 mL of 80% ethanol‐saturated sodium chloride solution and 250 mg of leaves were mixed by ultrasound for 30 min. The extraction solvent was discarded after centrifugation; 15 mL of 40% perchloric acid was added and mixed. The color reaction of starch was determined by iodine method at 570 nm by using colorimeter.

### Protein extraction, quantification, digestion, purification, and fractionation

As reported in previous literature by Lu [25], samples at each time point were used to extract, quantify, digest, and purify protein. The pooled desalted peptide sample (100 μg desalted peptides of each sample, 13 samples) was divided into eight fractionations and dried by Speedvac.

### Reference library generation and SWATH quantitative analysis

The eight fractionations were detected respectively by mass spectrometer TripleTOF 5600+ (AB SCIEX, USA) combined with Ekspert^TM^nanoLC 415 (AB SCIEX, Framingham, MA, USA) in information‐dependent acquisition (IDA) mode. The liquid phase systems were consisted of mobile phase A (0.1% formic acid in water) and B (0.1% formic acid in ACN). The peptides were loaded onto a nanoLC trap column (ChromXP C18‐CL, 3 μm, 120 Å, 350 μm × 0.5 mm, AB SCIEX). Afterward, a nanoLC column (75 μm × 15 cm, 3C18‐CL‐120, 3 μm, 120 Å, AB SCIEX) with a flow rate of 300 nL·min^−1^ was used to elute peptides by employing a nonlinear 120‐min gradient: buffer B increased from 5% to 8% in 0.5 min, 22% in 40 min, 22% to 30% in 80 min, 30% to 50% in 95 min, 50% to 80% in 105 min, 80% in 106 min, 80% to 5% in 112 min, 5% in 120 min. The mass tolerance was set to 50 mDa, and the detection limit was set to 100 cps. A maximum of 40 precursors per cycle were chosen and the mass range was 350–1500 *m/z*. The MS data of all eight fractionations were searched against the *N. benthamiana* database (version v1.0.1) by ProteinPilot to generate a SWATH library. The following parameters were set: iodoacetamide cysteine alkylation, digestion by trypsin, no special factors, and false discovery rate (FDR) < 1.0%. The proteins, peptides, and their fragment ions were included in the library for extracting SWATH data.

Proteins of each time point were analyzed in SWATH‐MS mode, and the conditions of liquid phase were the same as those in IDA mode. The SWATH data acquisition was 60 dynamic windows and the precursor ion mass range was set at 350–1500 Da. The generated library and the SWATH data files were together imported into the software peakview (version 2.1.10, AB SCIEX), the target peptides were extracted after the mass spectrum peak calibration. For each peptide, five transitions were selected, and a peptide confidence level was set to > 99%. The ‘mrkvw’ file containing protein and peptide information was loaded into Marker View software (version 1.2.1.1, AB SCIEX) for quantitative analysis.

### Bioinformatics

WGCNA was used to find the relationship between biological data and specific biological samples. Genes with similar expression patterns were grouped into the same module. Modules with high correlation which were merited deeper study can be found by calculating the correlation coefficients between module eigengenes and different time points.

The complete sequences of identified proteins were searched against the *N. benthamiana* database (version v1.0.1) and generated as ‘.fa’ files, which were loaded in the multiple sequences in string software (http://string‐db.ory/, version 10.5) and the *Arabidopsis thaliana* was selected as the organism. The confidence of protein analysis was set as high (> 0.900) and the results were output in ‘string_interactions. tsv’ and ‘enrichment KEGG. tsv’ files. Cytoscape (version 3.7.1) was used to visualize the PPI network by importing the network from ‘string_interactions. tsv’ file, proteins were classified based on KEGG results. A continuum of orange to blue was chosen as label color (from orange to blue, the protein’s degree value was getting higher). The proteins with high degree were considered as hub proteins in the PPInetwork. *t*‐test ˂ 0.05, fold change ˃ 1.25 or fold change ˂ 0.8 were set to filtrate differentially expressed proteins and differentially accumulated metabolites.

Principal component analysis (PCA) and Hierarchical Clustering Alg (HCA) were performed on the whole samples using simca software (version 13.0.0.0, Umetrics, Malmö, Sweden).

## Results

### Plant phenotypes and analysis of proteome

**Fig. 1:**
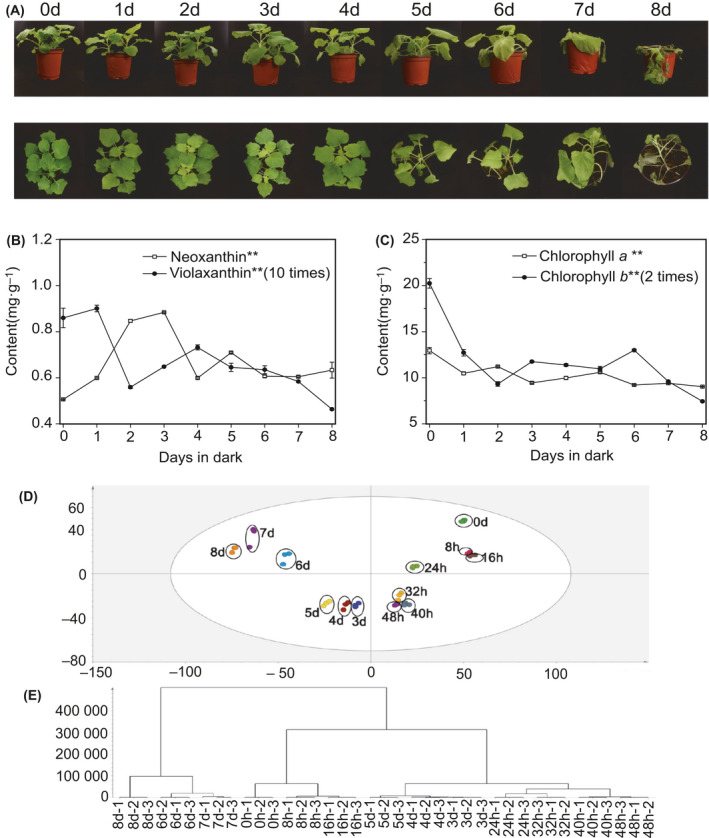
Phenotypic changes and PCA of proteome under dark stress. (A) Photographs of example plants taken on each of 8 days of dark treatment. Side view and top view. (B, C) Changes in the concentration of neoxanthin, violaxanthin, chlorophyll a, and chlorophyll b in leaves. Error bars indicate standard deviation (SD) (*n* = 3). Analysis of variance (ANOVA) was used for statistical analysis. (‘*’: *P* value < 0.05, ‘**’: *P* value < 0.01). (D) PCA of SWATH data from both dark treatment groups and control (control = 0 day (0 h)). (E) Clustering dendrogram of samples obtained on the consensus correlation.

The senescence of dark‐treated *N. benthamiana* plants was examined. There was no visible yellowing in the first 4 days of dark treatment (Fig. 1A). After 5 days of dark treatment, the leaves exhibited a highly chlorotic phenotype compared to the control groups. The leaves began to lose pigment and showed structural collapse. After 6 days, the ratio of yellow plants increased. After 7 days, the leaves were constantly yellowing and exhibited remarkable collapse. As the dark treatment continued, the plants were finally withered and almost died. There were obvious changes in plastid pigment levels in dark‐treated plants, and four pigments were tested (Fig. 1B). The content of neoxanthin showed a bimodal change. There were significant increases on 3 days and 5 days under dark condition. The level of violaxanthin reduced rapidly on 2 days, with a slight increased at the next 2 days, and then steadily decreased from day 5. It was observed that all types of chlorophyll diminished during dark treatment (Fig. 1C).

Eight group samples obtained by fraction were used to generate the reference library, which contained 7320 proteins. The library was compared to a SWATH‐MS map with PeakView software, 5763 proteins were identified, with FDR ˂ 1% for both protein and peptide levels. The PCA model showed that all samples of control and dark‐treated groups are clearly separated (Fig. 1D), indicating that the proteins of dark‐induced plants have changed relative to the control plants. There were significant differences that accumulated through time in the dark‐induced plants. HCA performed a good correspondence between the protein clustering of each sample and the sampling time (Fig. 1E).

### WGCNA and PPI network of specific consensus modules

**Fig. 2:**
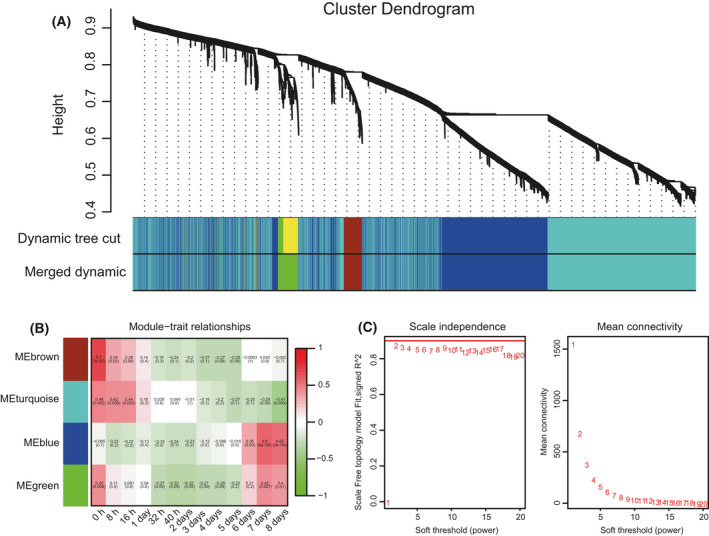
Weighted gene coexpression analysis diagram. (A) Dendrograms of sample proteins, with dissimilarity based on topological overlap, together with assigned module colors, showing four coexpression modules identified in all samples by WGCNA. The size of modules depended on the number of proteins contained in each module. (B) Module–trait relationships between consensus module eigengenes and the trait time. Each row corresponds to a module eigengene, each column to a time. Correlation coefficient and *P*‐value is shown in each cell. Red color represents a positive correlation, while green color means a negative correlation between a specific module and the trait time. (C) The soft thresholding power for a scale‐free network.

Using the WGCNA approach, the expression values of the 5763 proteins were used to construct the coexpression module. Four unique modules were identified in our data (Fig. 2A). Coregulated genes in each module are similar in their expression patterns. In WGCNA, correlated expression profiles indicate that there might be interactions among genes in the related pathways. For all samples, two modules had a high correlation with the time‐point samples: The turquoise‐colored module was positively associated with 8 h (*r* = 0.42, *P* = 0.008) and 16 h (*r* = 0.44, *P* = 0.05), and the blue‐colored module was positively correlated with 7 days (*r* = 0.6, *P* = 5e−05) and 8 days (*r* = 0.55, *P* = 3e−04) (Fig. 2B). One of the most important parameter was power value which mainly affects the independence and the average connectivity degree of coexpression modules. The appropriate power value (β = 2) was screened out (Fig. 2C), and the independence degree was up to 0.9.

**Fig. 3:**
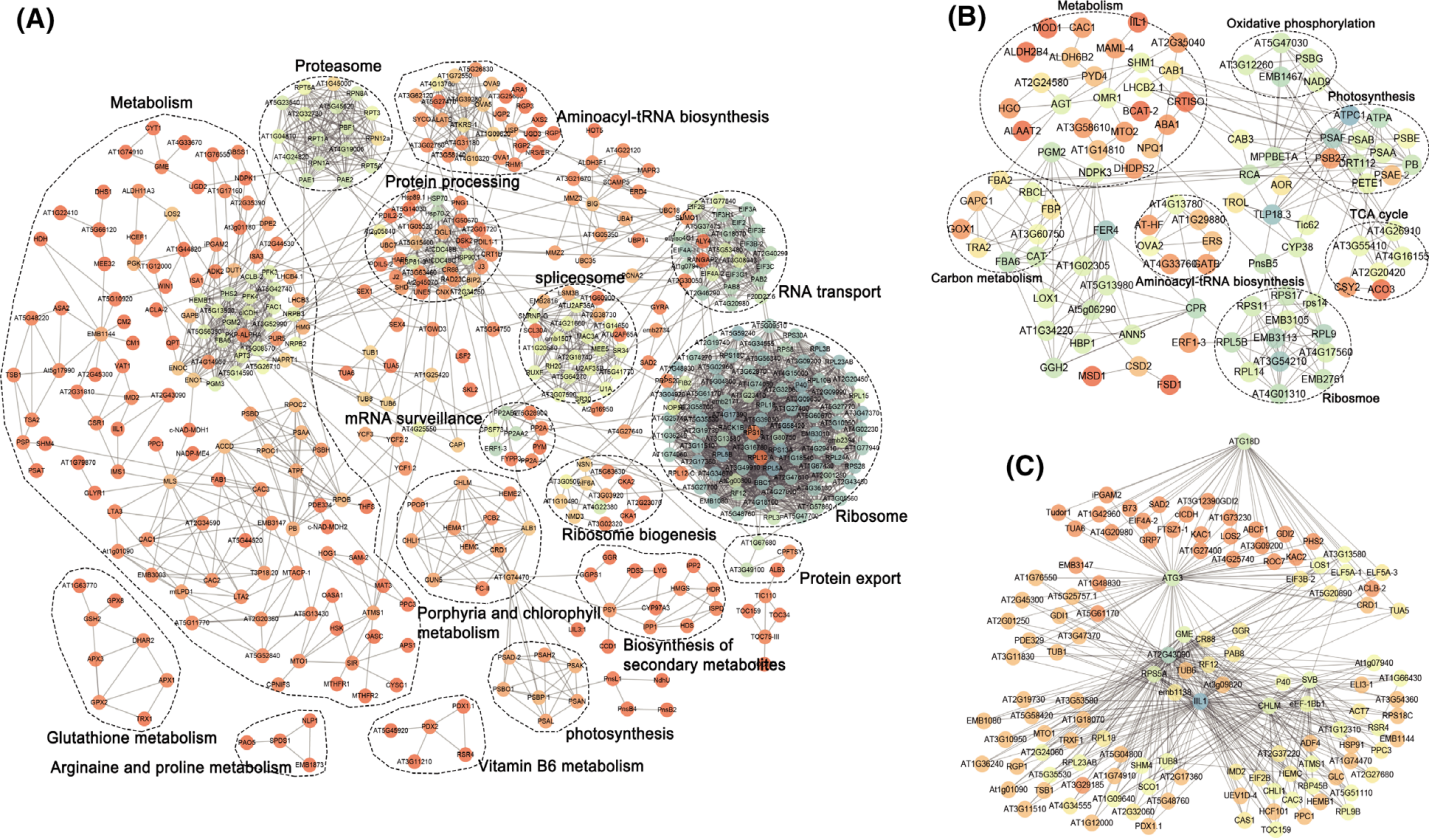
PPI networks. (A) The PPI network of turquoise module. (B) The PPI network of the blue module. (C) Predicted‐PPI network of autophagy‐related proteins in the turquoise module. Colors range from orange to blue; the degree score of corresponding protein was higher.

The network of PPI from the turquoise module proteins was established by string software (Fig. 3A). The network involves proteasome, RNA transport, protein processing, porphyrin and chlorophyll metabolism, spliceosome, and mRNA surveillance. The proteins of the blue module were also used to establish a PPI network that contained proteins at several key hubs (Fig. 3B). These proteins are mainly involved in carbon metabolism, oxidative phosphorylation, ribosome, photosynthesis, aminoacyl‐tRNA biosynthesis, and the TCA cycle.

Meanwhile, some proteins in the turquoise module confirmed to be highly associated with autophagy process (such as ATG proteins), and the proteins which have high weight value with ATG proteins in the turquoise module were selected and visualized using Cytoscape to construct a predicted‐PPI network (Fig. 3C).

### TEM and PCR analysis of *N. benthamiana* mesophyll cells under dark treatment

**Fig. 4:**
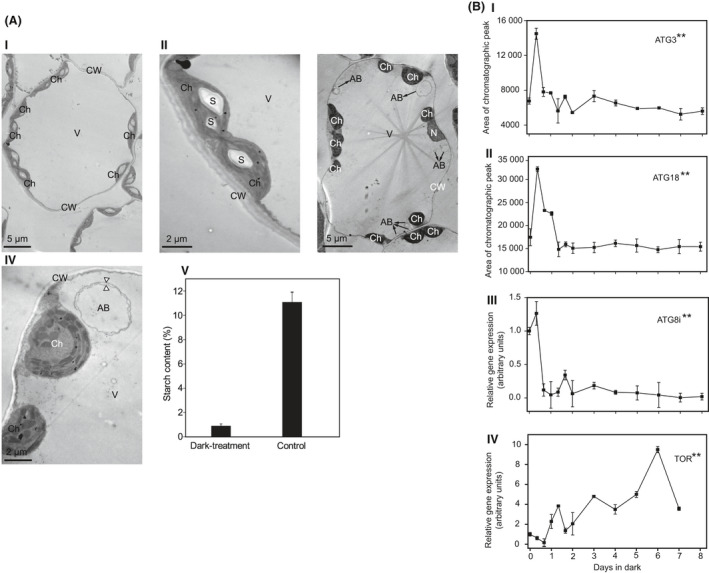
(A) Electron microscopy analysis of *N. benthamiana* mesophyll cells grown in control condition (I, II) or in dark stress (III, IV) after 4 days. (I) and (II) Control cell. The cytoplasm contains a vacuole and many chloroplasts full of starch grains. (III) and (IV) Dark‐treated cell. The amount of starch granules is reduced, and typical double‐membrane‐bound autophagic vacuoles appear in the cytoplasm. White arrows point to the inner and outer membranes of the autophagosomes in (IV). (V) Starch content of control samples and plants under dark condition for 4 days. Error bars indicate SD (*n* = 3). Ch, chloroplast; CW, cell wall; V, vacuole; S, starch; N, nucleus; AB, autophagic bodies. Scale bar: 5 µm (I, III), 2 µm (II, IV). (B) (I) and (II) Measurement of proteins ATG3 and ATG18 using SWATH‐MS, where the area of chromatographic peak indicates the changes in protein content. (III) and (IV) Relative expression of *ATG8i* and *TOR* measured by real‐time RT‐PCR. Error bars indicate SD (*n* = 3). ANOVA was used for statistical analysis. (‘*’: *P* value < 0.05, ‘**’: *P* value < 0.01).

TEM was performed to observe the changes of *N. benthamiana* leaf cells under dark stress (Fig. 4A). It was obviously observed that various typical double‐membrane autophagosomes (Fig. 4A III and IV) have accumulated in the dark‐treated plants, while these structures were not seen in the control plants (Fig. 4A I and II). The starch in chloroplast was distinctly decreased in the leaf cells after dark treatment (Fig. 4A III and IV) compared to the control (Fig. 4A I and II), which was consistent with the result of starch content detection (Fig. 4A V). These results indicated that dark stress could induce autophagy, which were consistent with the previously reported literature [3, 7, 8].

The expressions of ATG proteins (such as ATG3 and ATG18) were also investigated. The expression of ATG3 and ATG18 (Fig. 4B I and II) increased with a maximum at 8 h after dark treatment and then decreased. ATG8 plays an important role in autophagy [8, 27, 28] but was not detected by MS. We speculated that the level of ATG8 protein was too low to be detected by MS and employed the RT‐PCR to obtain the *ATG8i* gene expression of each time point. The results indicated that the *ATG8i* gene exhibited higher relative expression levels during the first 8 h of dark treatment and decreased sharply in the next 8 h and then remained stable (Fig. 4B III).

Target of rapamycin is an evolutionarily conserved Ser/Thr protein in eukaryotes. It is the main coordinator of nutrition, energy, and stress signal transmission networks [29, 30]. It retains the negative regulatory effect on autophagy in plants [31, 32, 33]. Similarly, the presence of TOR was not found by MS, so TOR was detected by PCR. The expression of *TOR* gene in dark‐induced *N. benthamiana* leaves was shown in Fig. 4B IV. In the first 16 h of dark treatment, the *TOR* gene continued to be downregulated, with the extension of the dark treatment time, its expression level was in an upregulated state relative to the control group. The expression of *TOR* gene was downregulated at 8 h; however, the ATG proteins/gene showed an upregulation at the same time, suggesting that autophagy was activated by TOR. Subsequently, the expression of *TOR* was upregulated and *ATG* was downregulated, confirming the negative regulatory effect of TOR on autophagy.

### Metabolite reprogramming under dark treatment

**Fig. 5:**
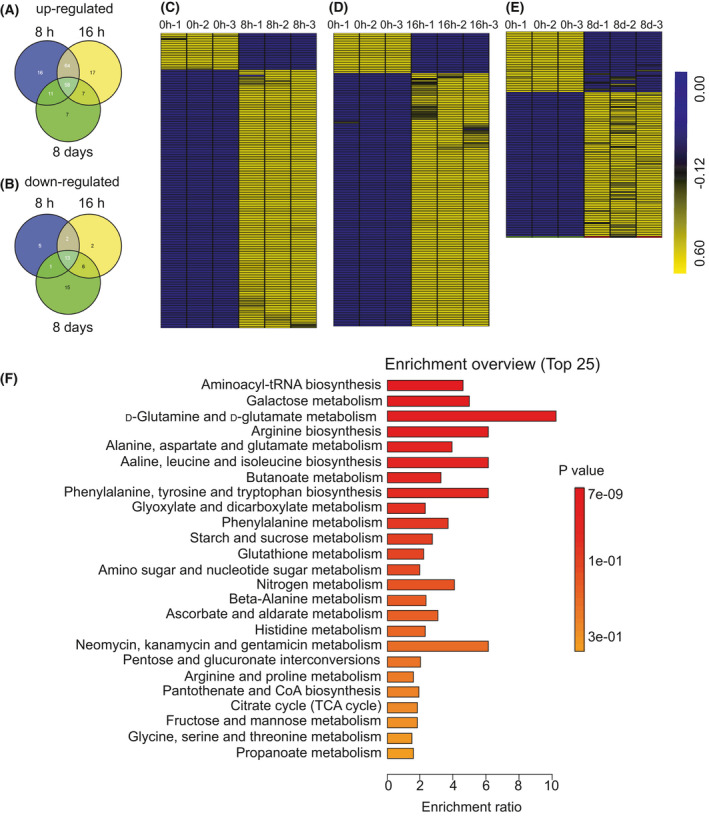
Analysis of identified metabolites. (A, B) Venn graph for upregulated/downregulated differentially accumulated metabolites between dark‐treated samples for 8 h, 16 h, and 8 days. (C–E) Heatmaps of differentially accumulated metabolites in control group, 8 h, 16 h, and 8 days under dark treatment. (F) Top 25 enriched pathways for differentially accumulated metabolites in all samples.

Based on the results of WGCNA, we mainly analyzed the metabolites of samples after dark treatment for 8 h, 16 h, and 8 days. 210 primary metabolites were detected including phenol, sugars, amino acids, nucleotides, organic acids, and alkaloids through GC‐MS. Compared with the control group, differentially accumulated metabolites were observed for 149 upregulated and 21 downregulated in the 8 h, 146 increased and 23 decreased in 16 h, 83 increased and 35 decreased in 8 days (Fig. 5A,B). Heatmaps of differentially accumulated metabolites were performed, and there were significant gaps between the dark‐treated samples and control group (Fig. 5C–E). A total of 183 differential metabolites were identified in all samples using a one‐way analysis of variance (ANOVA) (*P* < 0.05). metaboanlyst 4.0 (Xia Lab @ McGill, Ste. Anne de Bellevue, Canada) online software was used to perform an enriched pathway analysis for 183 differentially accumulated metabolites (Fig. 5F). ‘Aminoacyl‐tRNA biosynthesis’, ‘Galactose metabolism’, ‘D‐Glutamine and D‐glutamate metabolism’, ‘Arginine biosynthesis’, ‘Alanine, aspartase and glutamate metabolism’ and ‘Valine, leucine and isoleucine biosynthesis’ were highly enriched.

**Fig. 6:**
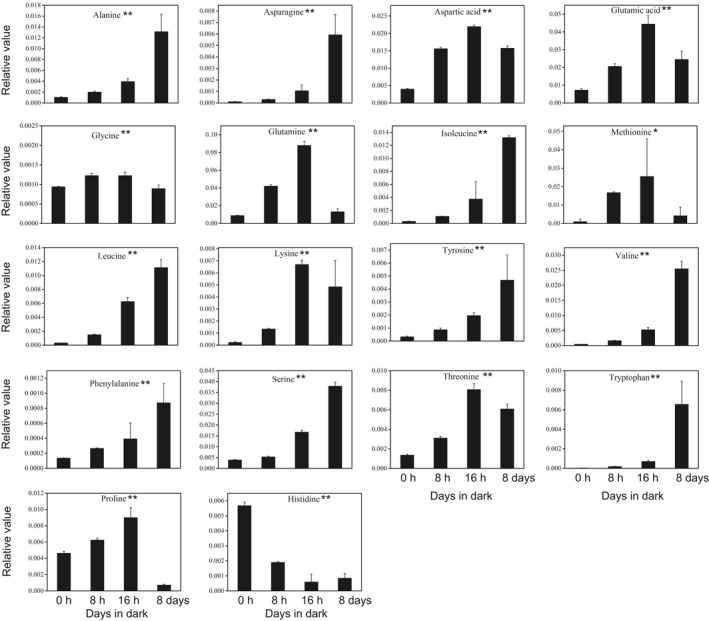
Changes of amino acid content in *N. benthamiana* leaves of the 0 h, 8 h, 16 h, and 8 days during dark treatment. Error bars indicate SD (*n* = 3). Analysis of variance (ANOVA) was used for statistical analysis (‘*’: *P* value < 0.05, ‘**’: *P* value < 0.01).

**Fig. 7:**
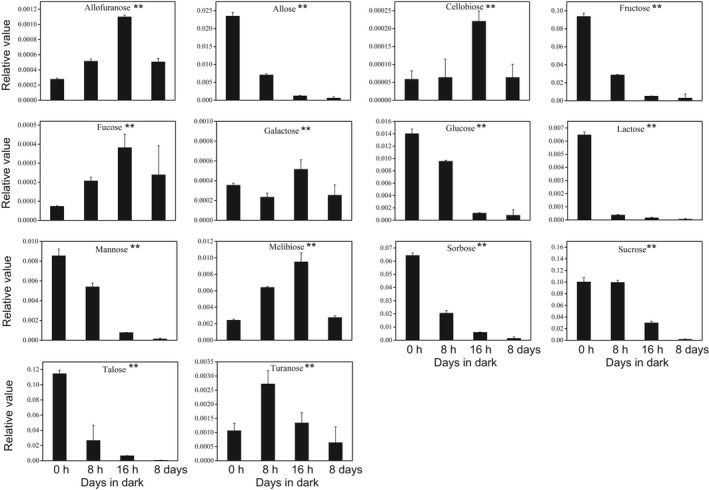
Changes of sugar content in *N. benthamiana* leaves of the 0 h, 8 h, 16 h, and 8 days during dark treatment. Error bars indicate SD (*n* = 3). ANOVA was used for statistical analysis. (‘*’: *P* value < 0.05, ‘**’: *P* value < 0.01).

Enriched pathway analysis indicated that amino acid and sugar metabolism played an important role in response to dark stress in *N. benthamiana*. Therefore, we observed the changes in amino acid and sugar levels during the dark treatment. As the dark period lengthens, most amino acids substantially accumulated except histidine, including those to be associated with senescence, such as phenylalanine, tyrosine, isoleucine, valine, and tryptophan (Fig. 6). During the course of dark‐induced carbon starvation, there were remarkable differences in sugar metabolism between dark treatment samples and the control group. The levels of allose, fructose, sorbose, talose, mannose, glucose, lactose, and sucrose reduced rapidly during extended dark period. There was an accumulation of allofuranose, fucose, melibiose, and cellobiose under the dark stress (Fig. 7).

### Changes in plant hormones and flavonoids under dark treatment

**Fig. 8:**
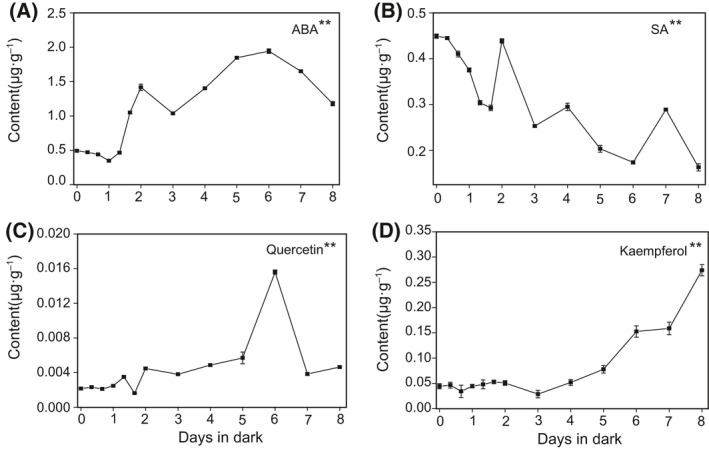
Changes in pigments, plant hormones, and flavonoids concentration in dark treatment samples through time. (A, B) ABA and SA in samples at different time points. (C, D) Changes of quercetin and kaempferol content in leaves. Error bars indicate SD (*n* = 3). ANOVA was used for statistical analysis. (‘*’: *P* value < 0.05, ‘**’: *P* value < 0.01).

The content of abscisic acid (ABA) showed an overall trend of increase during dark treatment and reached the maximum on 6 days (Fig. 8A). During the first 40 h of dark treatment, the content of salicylic acid (SA) was decreased. The contend showed a decreasing trend after a sudden increase from the 48 h to the 7 days following by a slight increase (Fig. 8B).

After 6 days of dark treatment, the content of quercetin showed an overall upward trend and reached the highest level on 6 days of dark treatment (Fig. 8C). The content of kaempferol did not change significantly in the first 4 days after dark treatment, but remarkably increased from the day 5 (Fig. 8D).

## Discussion

Connecting the expression of module proteins with specific experimental time points may help us to find the important modules under dark conditions. In the turquoise module (Fig. 3A), ribosome proteins had higher degree. Both ribosomal protein L24 (RPL24) and ribosomal protein L5A (RPL5A) belong to the assembly initiation proteins which can specifically bind rRNA and play roles in transcriptional regulation. Within protein processing, the HSP family proteins have been found to be essential for folding, assembling, reacting with, or degrading proteins damaged by stress. Members of HSP family proteins are fundamental in developmental processes and play various stress‐protective roles in plants [34, 35]. It was reported that HSP70 and HSP90 are crucial components of INF1‐mediated hypersensitive response in *N. benthamiana*, and they play a part in plant defense signal transduction pathway upstream or independent of the mitogen‐activated protein kinase (MAPK) cascade [36]. We found that the expression of HSP70 and HSP70‐2 proteins was respective downregulated, the content of chlorophyll decreased during dark stress. Referring to literature, the silencing of HSP70 lead to the significant increase of the chlorophyll degradation of *N. benthamiana* under stress [37, 38], which is consistent with our results.

Under comparison, some protein interactions were found both in the PPI network (Fig. 3A) and predicted‐PPI network (Fig. 3C) as expected, such as P40 and RPS5A, IIL1 and AT2G43090, RPS5A and RPL23AB, eEF‐1Bb1 and AT5G60390, and CHLM and HEMC. In the predicted‐PPI network (Fig. 3C), the two proteins with the highest scores were isopropylmalate isomerase large subunit 1 (IIL1) and isopropylmalate isomerase small subunit 1 (AT2G43090), which are the IPMI large subunit (IPMI LSU1) and small subunit (PMI SSU1), respectively. The phenotype of *Arabidopsis* is severely changed with blocked growth, narrow pale leaf with lacking chlorophyll, and abnormal flower shape when the *PMI SSU1* gene is knocked out [39]. The expression of IPMI SSU1 protein presented downregulation from 3 days after dark treatment, the leaves of *N. benthamiana* gradually turned yellow, and the content of starch was less in chloroplasts (Figs 1A and 4A). The results demonstrated that the PMI SSU1 play a vital role in normal growth and development in *N. benthamiana*. It is known that IPMI proteins are involved in the synthesis of leucine [40]; however, the level of leucine is not decreased but even increased with the strong reduction of IPMI proteins during the dark treatment. The accumulation of leucine may be due to the activation of autophagy, which cause protein degradation and recycling [29, 41], and the leucine could be as signaling molecule to regulate mTORC1 signaling pathway and autophagy; the autophagy is inhibited by the sufficiency of leucine [42]. Therefore, it is speculated that IPMI proteins may participate in the process of autophagy. ELF5A and RPS5A also showed the higher degrees. ELF5A protein has functions in RNA stability and the trafficking of RNA between the nucleus and the cytoplasm, or interacting with several proteins to participate the transport of RNA and proteins in cells [43]. ELF5A plays a role in regulating stress responds and development [44]. The absence of ELF5A leads to a delayed leaf senescence induced by dark stress and antiapoptotic phenotype of plants [45], which is consistent with our study, suggesting the *N. benthamiana* plants resisted the leaf senescence by reducing the expression of ELF5A protein under dark stress. ELF5A has been proven that it could mediate lipidization and autophagosome formation of ATG8 family proteins by translating of ATG3 in mammals [46]. Therefore, we speculated that ELF5A protein may also be involved in autophagy in plants through a similar pathway. The genes of coding ribosomal protein are reduced when plants suffer from stress such as sugar starvation [47], and deficiency of ELF5A resulted in reduction of translation‐related proteins and some ribosomal subunits [48]. The expression of ribosomal proteins RPS5A was downregulated and the stagnation of plant growth during dark stress may be due to the translational suppression of specific cell cycle‐related mRNAs. The literature has confirmed that there is a protein interaction between RPS5A and TOR [49]. In our research, it was also found that RPS5A was interactive with ATG3 (Fig. 3C). So it was suspected that RPS5A may participate TOR pathway to response to autophagy in *N. benthamiana*. Meanwhile, Jung et al. [50]. also support coordinated ubiquitination and deubiquitination activities can finely balance the level of regulatory some ribosomal proteins mono‐ubiquitination in autophagy processes. However, the specific mechanism is unclear and needs further research.

In the blue module (Fig. 3B), ATP synthase CF1 alpha subunit (ATPA) and photosystem I subunit F (PSAF) have a higher degree so that they can be considered top proteins belonging to the photosynthesis pathway. ATPA is the ATP synthase CF1 alpha subunit encoded by the chloroplast proteins ATPA, and PSAF is one of the PSI subunits and encodes subunit F of photosystem I [1]. Thylakoid lumen 18.3 kDa protein (TLP18.3)’s degree is very high, indicating that it occupies an important position in the blue module. TLP18.3 encodes a thylakoid lumen protein that regulates the repair cycle of photosystem II. TLP18.3 forms the polysome with photosynthetic apparatus and acts as a novel acid phosphatase in the thylakoid lumen. The pH of the thylakoid lumen decreases to strong acidic under stress conditions, which provide an activated environment for TLP18.3 removing the phosphate group from pSer or phospho‐Thr (pThr) of damaged proteins [51, 52].

SnRK1‐interacting protein 1, Tap46, and GDH belonged to blue module and were differentially expressed proteins. Studies have shown that SnRK1 may be involved in controlling and coordinating carbohydrate and nitrate metabolism to promote plant growth by regulating the transcription of several genes of sucrose synthase/sucrose degradation and participating in starch biosynthesis [53, 54, 55]. It has been speculated that SnRK1 positively regulated autophagy when plants were under stress and senescence [56]. For example, the *ATG8* gene could be induced by the overexpression of SnRK1 in the leaves of *Arabidopsis* [57]; the expression trends of SnRK1 were consistent with *ATG8i* in our data. In our study, the expression of SnRK1‐interacting protein 1 was significantly upregulated at 8 h under dark treatment which was consistent with the ATG8 conjugate encoded by *ATG* genes upregulated instantaneously by starvation. We speculated that the stress response in *N. benthamiana* occurred at 8 h during dark treatment, and autophagy was induced by increasing the expression of SnRK1‐interacting protein 1 to cope with carbon starvation under dark stress.

The target of rapamycin (TOR) is a ptdins3k‐related kinase which can regulate cell growth and metabolism in response to environmental changes, such as dark stress [58]. The TOR pathway was found to regulate the autophagy pathway in plant cells [33]. Some downstream proteins, including Tap46, have also been identified in the TOR pathway in plants [59]. Tap46, a regulatory subunit of protein phosphatase 2A (PP2A), can be dephosphorylated due to TOR signaling inactivation in nutrient‐deficient conditions. Tap46 and PP2A are essential in the TOR signaling pathway, such as promoting the translation of proteins, inhibiting autophagy and programming cell death, and Tap46 could be a negative regulator for autophagy [60, 61]. In this research, Tap46 was upregulated at 16 h after dark treatment, and the relative expression of *ATG8* was decreased, suggesting the overexpression of Tap46 may inhibit the processing of *ATG8* and formation of autophagosomes under dark stress.

Glutamate dehydrogenase (GDH) has important effects on the metabolism and transport of organic carbon and nitrogen, and it can be used as an indicator to evaluate the carbon/nitrogen status of plants [62]. The senescence process of tobacco leaves is usually accompanied by protein hydrolysis, increased ammonia content, and triggering the expression of GDH [63]. Previous studies revealed that the increase in GDH activity and content during pollen development in grapes may be related to the appearance of multiple vesicles which are similar to autophagic vesicles. Such multiple vesicles also appear abundantly in the case of carbon/nitrogen starvation in plants, and the number of vesicles corresponds to the autophagy activity in vacuoles [64, 65]. We observed the mesophyll cells of the treatment group and the control group by TEM and found that autophagosomes with double‐membrane structure appeared in the mesophyll cells treated with darkness for 4 days, the expression of GDH was significantly increased at 4 days. These results indicating that the increase of GDH expression may be related to the formation of autophagosomes during dark treatment in *N. benthamiana*.

Dark stress can affect plant amino acid metabolism. Engqvist et al. [66] and Barros et al. [67] determined the amino acid level of *Arabidopsis* wild‐type plants grown in dark environment and found that the content of asparagine, glutamic acid, isoleucine, valine, lysine, serine, tyrosine, phenylalanine, and threonine showed varying degrees of increase during the dark treatment process, which is consistent with our research results. Therein, valine, leucine, and isoleucine are collectively referred to as branched chain amino acid (BCAA). Previously, BCAA, aromatic amino acids, and lysine have been used as alternative substrates to maintain respiration during energy limitation [68]. Autophagy is induced by dark stress and transport proteins to vacuoles for degradation. Carbon starvation leads to increased protein degradation, releasing free amino acids as an alternative substrate for mitochondrial respiration, and promoting the synthesis of ATP through a pathway different from the classical respiratory pathway to compensate for the decrease in photosynthesis. This may be one of the possible reasons for the augment in amino acid content of *N. benthamiana* plants under dark stress [69, 70]. The accumulation of valine and isoleucine provide electrons to the mitochondrial electron transport chain in condition of dark‐induced carbon starvation, while the increase of phenylalanine and threonine could act as precursors for the synthesis of a broad spectrum of secondary metabolites with multiple biological functions and health‐promoting properties [71]. TORC1 is a highly conversed macromolecular complex composed of TOR kinase and other scaffold proteins to regulate the processes of protein synthesis and autophagy. In our study, isoleucine, phenylalanine, and TOR were upregulated with the extended darkness. Isoleucine and phenylalanine have been proved to inhibit autophagy by positively regulating the activity of target of rapamycin complex 1 (TORC1) in mammal and yeast [72, 73, 74].

Sugars are essential in energy metabolism, pathway signaling, and autophagy [21]. Studies confirmed that the content of carbohydrates decreased during dark treatment, including fructose, glucose, galactose, mannose, sucrose, and so on, which coincide with our results [66, 67, 75]. Tcherkez et al. [76] deemed that increased fatty acid oxidation activity leads to sugar starvation in plants under dark stress, and sucrose starvation could induce autophagy in *Arabidopsis* suspension cells. Although the content of trehalose in plants is low, it can act as a signal molecule to participate in the regulatory process of plants in response to stress [77]. Using transgenic technology to overexpress trehalose phosphate synthase to produce high level of trehalose, which can effectively improve the drought resistance of tobacco plants [78]. Autophagy was triggered by trehalose through the mediation of mTOR and enhanced by the overexpression of mictotubule‐associated protein light chain 3II (LC3II) which was regulated by trehalose and closely related to the number of autophagosomes in human [79]. The increase in trehalose content indicated trehalose played a vital role in maintaining cell membrane homeostasis, improving stress resistance, and participating in the process of autophagy during dark stress in *N. benthamiana*.

The degradation and breakdown of chloroplast is closely related to dark stress [80]. Nutrients are mainly stored in chloroplasts as photosynthetic proteins, such as ribulose bisphosphate carboxylase oxygenase (Rubisco). Studies shown that chloroplast degradation by autophagy has become a new way to reposition nitrogen in starved plants, and the Rubisco is encapsulated by autophagosomes and transported to vacuoles for degradation [81, 82].

Plant hormones regulate the normal growth of plants and could be easily affected by the external environment, including dark treatment. Phytohormones ABA can boost leaf senescence through complicated physiological processes [83]. ABA, the stress hormone, acts as the central medium of several stress responses in plants [84, 85]. The level of endogenous ABA increased by upregulating the expression of ABA biosynthetic genes *nine‐cis‐epoxycarotenoid dioxygenase 3* (*NCED3*) and *NCED5* under shade condition [86]. Prolonged shade treatment results in diminished branching because of accumulation of ABA [87]. One recent study proposes that the augment of ABA leads to leaf senescence and accumulation of reactive oxygen species (ROS) which may cause serious damage to lipids, proteins, and DNA in plants. Meanwhile, autophagy is triggered to degrade excessive ROS to help protect cells, ABA participate in a TOR‐dependent route of autophagy regulation [88, 89]. SA has been considered as a signal molecule of plants under abiotic stress [90, 91]. In *Arabidopsis* autophagy mutants, the content of SA was higher than that of wild‐type plants, and the mutant plants exhibited an enhanced senescence [92]. It has been reported that the efficient activation of the SA signaling pathways is dependent on light signals and the expression of SA‐induced gene is significantly decreased in dark‐induced senescence. Although the SA pathway does not express in dark‐induced senescence and the deficiency with SA signal leads to delayed senescence, the senescence proceeded normally in dark‐induced plants [83]. A number of researches shown that flavonoids had plenty of biological activities, such as antioxidant property [93, 94]. Under abiotic stress, the augment of quercetin/kaempferol content was related to the stress resistance of plants. After 6 days of dark treatment, the reduction of quercetin content may be caused by its antioxidant function to eliminate excessive ROS and resist adversity [95]. It was reported that increased ROS can induce autophagy [96]. Therefore, we speculate that quercetin and kaempferol may participate in the regulation of autophagy.

## Conclusions

In conclusion, this study uncovers the proteome and metabolome changes of *N. benthamiana* under dark stress. Through the analysis of PPI networks, HSP70, SnRK1‐interacting protein 1, Tap46, and GDH might shed new light on the roles in maintaining the dark acclimated condition via complex regulatory networks. IPMI, ELF5A, and RPS5A are speculated to be potential proteins for regulating autophagy under dark stress. TLP18.3 might play an important role in maintaining cell homeostasis during dark treatment. Our results expand the understanding of the complex regulatory mechanisms against dark stress in plants.

## Conflict of interest

The authors declare no conflict of interest.

## Author contributions

QS and P‐PL designed the project. J‐JS, Q‐SC, and Q‐XZ performed experiments. J‐JS, Y‐LX, Z‐FL, H‐NZ, and H‐YM analyzed the data. J‐JS and P‐PL wrote the manuscript. QS and P‐PL reviewed and edited the manuscript.

## Supporting information


**Table S1.** List of all identified *N. benthamiana* proteins’ annotation and expression (The expression value corresponds to the chromatographic peak area).

**Table S2.** List of AGI codes in Figure 3A which are supposedly orthologs of the identified *N. benthamiana* proteins.

**Table S3.** List of AGI codes in Figure 3B which are supposedly orthologs of the identified *N. benthamiana* proteins.

**Table S4.** List of AGI codes in Figure 3C which are supposedly orthologs of the identified *N. benthamiana* proteins.

**Table S5.** List of all identified GC‐MS components from *N. benthamiana* (The values for the different time points correspond to the relative value).

**Table S6.** List of discussed the identified N. benthamiana proteins in the text specifically (Relative expression of ATG8i and TOR measured by real‐time RT‐PCR, the others are protein expression).

**Appendix S1.** The genome location of *ATG8i* and *TOR* primer hybridization.

## Data Availability

The data that support the findings of this study are available from the corresponding author upon reasonable request.
