# Supplementary material for: Proteomic and metabolomic analysis of Nicotiana benthamiana under dark stress
Source: FEBS Open Bio. 2021 Dec 16;12(1):231–49. doi: 10.1002/2211-5463.13331 (PMC8727940; doi:10.1002/2211-5463.13331)
Supplement: Supplementary file 7 — Appendix S1. The genome location of ATG8i and TOR primer hybridization. [file FEB4-12-231-s007.docx]

Appendix S1

The eukaryotic translation elongation factor 1 alpha 1 (EF1α) was used as reference gene. The forward primers of EF1α were 5'-TGGACACAGGGACTTCATCA-3' and reverse primers of EF1α were 5'-CAAGGGTGAAAGCAAGCAAT-3'

ATG8I

>Niben101Scf01773g03004.1 sp|Q9LRP7|ATG8I_ARATH *-*- Autophagy-related protein 8i IPR004241 (Autophagy protein Atg8 ubiquitin like), IPR029071 (Ubiquitin-related domain)

TGGTGACACACTTTGGTGAACTATAAAAACTGAAGGGTCTGAGAAAAAGAGAACTGCGGTGAACGTGGATCGAACACGTGACCTTCAGATCTTCAGTCTG

ACGCTCTCCCAGCTGAGCTATCCCCGCAAGTTGTTGTGAAGCTCTTCTAGTGTATATAATTATTCTTCGATTAAGAACGTCTTTTCCACGTATTCTTATGATCTTATCTTTAAGGACAGAAACATTCATCCATAGACGGGTCGGTTGTGAAGCAGGGAGATGGGGAAGGCTTTCAAAAAAGAATTTTCAGACGATGAGAGACTCGCAGAATCTCAAGATATAATCGCCAAATATCCTGATCGACTGCCGGTGGTGGTTGAAAGATATTCAAAGACTGACCTTCCTGAGATGGAAAAGAAGAAGTACCTGGTACCCCGTGATATGTCCGTTGGCCAATTTATCCACATTCTGAGTGGCAGACTCCATCTGGCTCCTGGGAAAGCTCTCTTCATGTTTGTGAATAACACCTTGCCTCAAACAACAAGCTTGATGGAGACGGTGTATGATTCTTTCAAGGATAAAGATGGGTTCCTCTACATGTGCTACAGCAGTGAGAAAACCTTTGGTCGTGCAAATAGTTGAGCATATTGTCATTCCTAGGAAGTGCAACACATTGTGAACTAAAATATGTAAATGGTATCTGTATTGCATGTCCGGCATACCTGTAAATTCTTGCACTAGGGTGTCTATACTCTCAACTGTATATCTGAAGGAAATTCAAATGTTATTCTATCGTCCCTTGGAAAGAATAAAGAATACAAAGGTTGCAGTAAGTTTAAGGGTTAGGCCATTGTCTCGAACTAGAAGTTTTATTTTGTATCTCATACAACCAACACTAGTTGTATGAGGTTTCTTTTTGTCTCACAAATTTGTGGACCTCAATCTCACACTTCTGAGTCCATAAAAGTCTGAATTCACAAATTCGTGAGACAAAAAGGAGCCTCATACCACTAGTGTCAGTGATATGAGAGATAAAATTTTCTCCCTCGAACAGTTGAGACAGTCCTTGTAGTTGGTGATAAATAATCCTAATTTTTTCTCCAATTCCTAATCTATTGAATTGAGAAGCCAAAAATTTCCGTGGTAAATTTTGAGAGTGCAATTGCTAATATAGACCCTTAGCCAG

5' ATATAATCGCCAAATATCCTGATC 3' upstream primer

5' TGTTGTTTGAGGCAAGGTGTTA 3' downstream primer

The autophagy-related protein 8i (ATG8i) genome sequence is shown above, our primer hybridization take place from 318 to 341 bp (upstream primer) and 502 to 523 bp (downstream primer) with yellow color marks. The total qPCR product is 206 bp in length with red line marked.

TOR:

>Niben101Scf00953g08007.1 AT1G50030.1 IPR009076 (Rapamycin-binding domain), IPR014009 (PIK-related kinase), IPR016024 (Armadillo-type fold), IPR024585 (Domain of unknown function DUF3385, target of rapamycin protein)

ATGGCTACCACCAGTCAGGCGATCCGTTATCCAGTTGCAACCACCGGTGCCGGAAATATTGATGCTCTCAATAGAGTTCTTGCTGACCTCTGCACCAGAGGCAATCCTAAGGATGGAGCTACATTGGCCTTGAGGCGGCTCGTAGAGGAAGAAGCTCGTGATCTCAGTGGAGAAGCTTTTGCTCGTTTTATGGATCATCTATATGAACGTATTACTACATTTCTTGATAGTAATGAAGTTTCTGAAAATCTGGGAGCATTGAGGGCTATTGATGAGCTAATAGATGTCACCATCAGTGAAAATGCATCAAAAGTGGCAAAATTCTCCAATTACATGCGAGCTGTTTTTGAAACAAAGCGTGATCGTGAAATCTTGGTCCTTGCTAGTAAAGTTCTGGGTCACCTAGCTAGATCTGGCGGTGCAATGACTGCAGATGAAGTGGAACGTCAGGTAAAAGTTGCACTAGGATGGCTTCGTGGTGAAAGAATTGAGTATCGTTTCTTTGCTGCCGTCTTAATATTAAAGGAAATGGCGGAAAATGCTTCAACTGTTTTCAATGTTCATGTGCCGGACTTTGTGGAGGTTATTTGGGTTGCTCTGAAGGATCCAACATTGGCTGTTCGAGAGAAGGCTGTCGAGGCATTGCGTGCCTGCCTTCGCGTTATTGAAAAGCGCGAGACCCGATGGCGTGTTCAGTGGTATTATAAGATGTTTGAGGCTACCCAAGATGGATTGACCAGAACTGCGCCTGTTCATAGTATACATGGCTCCCTTCTCGCAGTGGGAGAGCTGCTAAGTTTTATGATAGGCTGTGATTTTGTTTTGCTGGAATTAGTTTCACCTTTCTTCATTAAAGCCCACGCTTTATTTGCGGTTGAGGCCCCAACAGACCTTAGAGCTTTTTTGCGCTTTTTGCCTTTGATAACACTGAAGGGTAGGCGCAAGTTGAATAATATGCTCAAGTCACATGATGTGGGAATTGGCCCCACTATTATCTGGATGTGGTATGATAAATGTTACCTGATGCATGCAGATTTCTTCATCAAAATGTGCAGGAATACAGGAGAGTTCATGATGTCAAGATACAGGGAGGTTGCGGAAATTGTTATAAGATACCTGGAGCACCGAGATCGCCTAGTTCGTCTCAGCATAACTTCTCTACTTCCTCGAATTGCCCATTTCCTGCGTGATCGATTTGTGACTAACTATTTAACGATATGCATGAATCATATACTTCATGTCCTTAAAATACCTGCAGAACGTGCCAGTGGGTTCATCGCTCTTGGGGAGATGGCTGGTGCTCTGGATGGTGAACTCATTAACTATTTGCCGACAATAACCTCTCACTTGCGTGATGCGATTGCTCCCCGCAGAGGCAGGCCCTCATTTGAGGCTCTGGCATGTGTTGGAAATATTGCTAAAGCAATGGGACCAGCCATGGAGCCTCATGTTCGTGGTCTCTTGGATGCTATGTTTTCTGCTGGGCTTTCCCTGACACTAGTGGAAGCCTTGGAGCAAATAACTGAAAGCATTCCATCTTTGTTGCCGACCATTCAAGATCGACTGCTTGAATGTATTTCAGCAATTCTCTCCAGATCTCATCATGCACTCTCAAGACAATCAACTGCTATGAGTCGAGGGCATATTGCAACAGTTATCCCCCAAGTACCAGAACTGAGTGGTCCTGCGCTAGTTCAACTTGCTTTGCAGACTCTAGCTCGTTTTAATTTCAAGGGCCATGATCTTCTTGAGTTTGCAAGGGAGTCTGTTGTTGTATATTTAGAAGATGAGGATGGAGCTACACGAAAAGATGCTGCGCTATGTTGCTGCAAACTAGTAGCAAATTCTTTCTTGGCGATGTCTTCTACCCAGTTTAGTCCTAGTAGAATCAATCGTGCCAGTGGAAAGCGACGTCGACTTGTTGAAGAGATTGTGCAAAAACTTCTCATTGCTGCTGTTGCGGACGCTGATGTTACTGTTCGGCATTCGATTTTTTCTTCTCTGTATGCTGATGGAGGATTCGATGAGTTTCTGGCTCAGGCTGATAGTTTGACAGCTATATTTGCCACTCTAAATGACGAGGATTTTGAAGTTCGTGACTATGCAATTTCACTAGCTGGTAGACTATCTGAAAAGAATCCAGCATATGTTCTTCCAGCACTTCGTCGCCATCTTATTCAGCTGTTAACTTACCTAGAACAAAGTGCAGATAATAAATGTAAAGAAGAGAGTGCAAAGTTATTGGGTTGCTTGATTCGCAATTGTGAACGATTAGTTCTTCCATACATTGCTCCCATACACAAGGCTCTTGTTGCGAAACTCTGTGAAGGCACAGGAGTCAATGCGAATAGTGGCATTATTAGTGGAGTTCTAGTGACTGTTGGAGATCTTGCCAGAGTGGGTGGCTTTGCCATGCGGCAGTATATTTCAGAACTTATGCCATTAATCGTTGAAGCTCTACTGGATGGGGCAGCTGCCACGAAACGGGAAGTGGCCGTTTCAACACTTGGTCAAGTTGTACAGAGTACAGGATATGTCATAACTCCATACAATGAGTATCCTCAGTTGCTTGGGTTACTCTTGAAACTGCTTAATGGTGAACTGGCTTGGTCAACCAGAAGAGAGGTTTTGAAGGTTCTTGGCATCATGGGTGCGTTAGATCCCCATGTGCACAAGCGCAATCAGCAAAGCTTACCTGGATCCCATGGTGAAGTTACCCGGGTGACCGGTGATCCTGGTCAACATATAAGATCAATGGATGAATTGCCTATGGATCTCTGGCCCTCCTTTGCAACATCTGAAGATTATTATTCCACTGTTGCTATCAACTCACTCATGCGGATACTCAGGGATCCATCTCTGTCAAGTTACCACCAGAAAGTGGTTGGATCTCTTATGTTTATTTTCAAGTCCATGGGCCTCGGCTGTGTCCCTTATTTGCCTAAGGTTTTGCCTGATCTCTTTCATATTGTACGAACATGTGAGGATGGTCTTAAAGAATTTATAACATGGAAGCTTGGAACCTTGGTATCTATTGTCCGCCAGCACATCCGTAAGTATCTGCCAGAATTGCTCTCTCTGATATCAGAAATATGGTCATCCTTCAGCTTGCCTGTTGCTAACAGACCTGTTCACATTGCTCCTATTCTGCATCTCGTGGAGCAACTTTGCTTGGCACTCAACGATGAGTTCAGAAAGTACCTTGCTGATATACTTCCCTGCTGTATTCAAGTTCTTACTGATGCAGAGAGGTTTAATGACTGCACATACGTTATTCCTATTCTCCACACGCTTGAAGTTTTTGGTGGGACATTAGATGAGCATATGCATCTGCTTCTTCCTGCACTTATTCGGTTGTTTAAATTGGATGCTTCAGTAGAAGTAAGACGCGGTGCAATCAAAACTCTCACAAGATTGATACCTCGTGTGCAGGTCACTGGACACATATCTTCTCTTGTGCATCACTTGAAGCTTGTCTTGGACGGGAACAAAGAAGAGCTCAGGAAGGATGCTGTTGATGCACTCTGTTGTCTAGCTCATGCTCTTGGAGAGGACTTCACCATTTTTATTCCTTCTATTCACAAGCTTATGGTTAAACATAGGCTGCAGCACAAGGAATTTGAAGAAATCCGAGGACGACTGGAAAAACGTGAGCCACTGATTTTGGGGAGCACCGCAGCTCAGAGATTAAATCGGCGGTTCCCGGTTGAGGTCATCAGTGATCCTTTGAGTGATGGAGAGAATGACCACTACGAGGTTGGGACGGACATGCATAAGCAGCTTAAAAGCCATCAGGTTAATGATGGCAGATTGCGTACCGCTGGTGAGGCTTCTCAACGAAGCACTAAAGAGGATTGGGCAGAGTGGATGAGGCATTTCAGCATTGAACTTCTGAAAGAATCACCTAGTCCAGCATTGCGAACTTGTGCAAGACTCGCTCAACTGCAGCCTTTTGTTGGGCGAGAGTTGTTTGCTGCAGGTTTTGTTAGTTGCTGGTCACAACTTAATGAGGCTAGTCAAAGGCAGCTAGTACGTAGTCTAGAAATGGCGTTTTCTTCTCCAAATATCCCTCCTGAAATTCTTGCTACACTTCTGAACTTGGCGGAGTTTATGGAACACGATGAGAGACCCCTTCCTATAGATATCCGTCTGCTTGGTGCTCTTGCGGAGAAGTGTCGAGCATTTGCAAAGGCCCTACACTACAAGGAAATGGAATTTGAAGGCGCACTTTCAAATAGGAGGGATGCAAATCCTGTTGCTGTAGTTGAAGCTTTAATCCATATAAATAATCAATTACATCAACATGAGGCAGCTGTTGGAATATTAACATATGCTCAGCAGCATTTGGGGGTTCAATTGAAGGAGTCATGGTATGAGAAATTGCAACGCTGGGATGATGCTCTTAAAGCATACACTGCTAAGGCGTCACAAGCTTCGAGTCCACATCTTGCTTTGGATGCTACTTTAGGGCGTATGCGATGCCTTGCTGCTCTAGCTCGGTGGGAGGAGCTTAACAATCTTTGTAAGGAATACTGGACGCCAGCTGAGCCAGCAGCTCGACTGGAAATGGCACCAATGGCTGCTAGTGCTGCCTGGAACATGGGTGAGTGGGATCAGATGGCAGAGTATGTTTCTCGGCTTGATGATGGTGATGAAACCAAACTCCGAGTCTTGGGAAATACTGCTGCCAGTGGTGATGGAAGTAGTAATGGCACCTTTTTCAGGGTCGTTCTTCTAGTTCGGCGAGGGAAGTATGATGAAGCACGTGAATATGTTGAAAGAGCAAGGAAATGTTTGGCGACCGAGCTCGCTGCACTGGTTCTTGAGAGCTATGAACGTGCTTACAGCAACATGGTCCGTGTTCAGCAGCTTTCTGAATTAGAAGAGGTGATTGAATACTGTACTCTTCCTATGGGAAACGCTGTTGCTGAAGGAAGAAGAGCTCTTGTTCGCAATATGTGGAATGAGCGCATAAAGGGTACAAAAAGAAATGTTGAGGTTTGGCAAGCACTTTTAGCTGTGAGGGCACTTGTATTGCCTCCTACAGAAGACATTGAAACATGGATCAAGTTTGCATCACTTTGCCGGAAGAATGGCAGAATTAGCCAAGCTAGATCTACATTGGTTAAACTTTTACAGTTCGATCCAGAATCAACTCCTGCAACTGTGCGGTATCATGGCCCCCCTCAGGTGATGCTAGCATACTTAAAGTACCAATGGTCACTTGGCGAGGATCATAAGCGAAAGGAAGCCTTTGCTAGGTTGCAGGACCTTGCCATGGACCTCTCAAGAACAGCAGCTCTTCAACCAGTAATGCAGAATGGATTAGTTGCTTCTTCTGGTGTGCCACTTGTTGCTCGTGTATATCTCAGACTCGGCACTTGGAAGTGGGCACTTTCTCCTGGTTTGGATGATGATTCTATACAAGAAATTCTTAGTGCATTTAGAAATGCTACTCACTGTGCAACGAAGTGGGGAAAGGCATGGCATACCTGGGCACTTTTCAATACCGCAGTGATGTCTCATTACACACTGAGAGGTTTTGCGAATATTGCTTCACAGTTTGTTGTTGCTGCCGTAACTGGTTATTTTCACTCTATAGCATGCGGAGCACATGCTAAGGGTGTTGATGATAGTTTACAGTTTTACTTTTCTAATCGTCGTGTTTCTGAGTTTCAGGATATTCTTCGTCTTCTTACTTTGTGGTTTAACCACGGAGCTACTTCGGATGTCCAAATGGCATTGCAGAAAGGATTCACACATGTTAACATCAACACATGGTTGGTTGTTTTACCTCAGATTATTGCACGGATACATTCAAATAACCATGCTGTCAGAGAGCTGATACAATCCTTGCTAGTGCGAATTGGACAGAGTCATCCACAGGCCCTTATGTATCCGCTTCTTGTGGCATGTAAGTCAATTAGCAATTTGCGCAGAGCTGCGGCTCAAGAAGTGGTCGATAAAGTTAGACAGCACAGCGGCGTACTCGTTGATCAGGCCCAACTTGTCTCAAAGGAGCTTATCAGGGTTGCGATACTGTGGCATGAAATGTGGCATGAGGCACTGGAAGAGGCCAGCCGTTTATATTTTGGCGAACACAACATCGAGGGCATGCTGAAGGTGTTAGAGCCTCTGCATGAAATGCTTGAGGAAGGAGCAATGAGGAACAATACCACTATAAAGGAGAAAGCATTCATCCAGGCATACCGTCTTGAGTTGTTGGAGGCCTATGAATGTTGTATGAAGTATCGGAGAACTGGTAAAGATGCTGAATTAACGCAGGCTTGGGATCTCTATTATCATGTATTCAGGCGGATAGATAAGCAGCTTCAAACACTCACAACCCTGGATTTGCAGTCTGTTTCCCCAGAGTTATTGGAGTGTCGAAATTTGGAGCTAGCTGTTCCTGGAACTTATATAGCAGATGCACCAGTGGTGACAATTGCATCATTTGCACCCCAACTTGTTGTAATTACATCCAAACAACGGCCTCGAAAATTGACAATCCATGGGAGTGATGGAGAAGACTATGCCTTTTTGCTCAAAGGGCACGAAGATCTACGCCAAGATGAACGTGTCATGCAGTTGTTTGGTCTGGTTAATACTTTGCTCGAGAATTCTAGAAAGACTGCAGAGAAAGATTTATCAATTCAACGATATGCTGTCATTCCATTGTCCCCTAATAGCGGACTGATAGGATGGGTTCCAAATTGCGACACCTTGCACCAGCTTATTCGAGAATATAGGGATGCCCGGAAGATCACCCTAAATCAAGAGCATAAATTGATGCTGAGTTTTGCACCGGATTATGATAATTTGCCACTTATTGCTAAGGTGGAGGTGTTTGAATACGCTTTGCAAAATACAGAAGGGAATGACTTATCAAGGGTTCTTTGGTTAAAGAGTCGTACTTCTGAAGTCTGGCTGGGCAGAAGAACAAATTATACAAGAAGTTTGGCTGTCATGAGTATGGTTGGATACCTTCTTGGTTTGGGTGATCGACATCCTAGTAACCTCATGCTTCACCGATACAGTGGGAAGATTCTGCATATTGACTTTGGAGATTGCTTTGAAGCTTCAATGAATCGGGAGAAGTTTCCAGAGAAGGTTCCCTTTCGACTCACTAGAATGCTTGTAAAAGCAATGGAGGTTAGTGGTATAGAGGGAAATTTCCGGTCAACATGTGAGAATGTAATGCAAGTTCTCCGACTGCATAAAGATAGTGTTATGGCTATGATGGAGGCCTTTGTTCACGATCCACTTATAAATTGGCGTCTTTTCAACTTCAATGAAGTTCCGCAAATGTCCACACTTGCCAGTGCACATGTCCCTCCTGTTACGAACAGTGAGGAATCTTCTTCAAATAGAGAGCTTCTTCAGCCACAAAGGGGTGCAAGGGAGAGAGAACTGCTTCAGGTGGTCAATCAATTAGGTGATGCCAATGAGGTTCTAAATGAACGTGCTGTGGCTGTTATGGCTCGAATGAGTAATAAACTCACAGGACGTGATTTTACTGCTGCTTCTACATCTGCGAGCTCTCTACAACATGCACTGGACCACAGTACGTTAATTTCTGGAGAGACGCGTGAAGCTGATCATGGTTTATCAGTGAAACTACAAGTCCAAAAACTTATTCAACAAGCGTCGTCTCATGAAAATCTTTGCCAAAATTATGTTGGGTACGATCCAGATTCCCAGGATAACCTCTCTCTCTCCCACCCTCCCTCTCTCTCTCTCTCTCTCTCTCTCTCTCTCATTGGATTGTTTCATTTGCTCTGA

5' AAGCCCACGCTTTATTTGCG 3' upstream primer

5' TTCAACTTGCGCCTACCCTT 3' downstream primer

The target of rapamycin protein (TOR) genome sequence is shown above, our primer hybridization take place from 854 to 873 bp (upstream primer) and 931 to 950 bp (downstream primer) with yellow color marks. The total qPCR product is 97 bp in length with red line marked.
